# Management Effectiveness of the World's Marine Fisheries

**DOI:** 10.1371/journal.pbio.1000131

**Published:** 2009-06-23

**Authors:** Camilo Mora, Ransom A. Myers, Marta Coll, Simone Libralato, Tony J. Pitcher, Rashid U. Sumaila, Dirk Zeller, Reg Watson, Kevin J. Gaston, Boris Worm

**Affiliations:** 1 Scripps Institution of Oceanography, University of California San Diego, La Jolla, California, United States of America; 2 Department of Biology, Dalhousie University, Halifax, Nova Scotia, Canada; 3 Institut de Ciènces del Mar, ICM-CSIC, Passeig Marítim de la Barceloneta, Barcelona, Spain; 4 Istituto Nazionale di Oceanografia e di Geofisica Sperimentale–OGS, Sgonico-Zgonik, Italy; 5 Peter Wall Institute for Advanced Studies, University of British Columbia, Vancouver, British Columbia, Canada; 6 Sea Around Us Project, Fisheries Centre, University of British Columbia, Vancouver, Canada; 7 Biodiversity and Macroecology Group, Department of Animal and Plant Sciences, University of Sheffield, Sheffield, United Kingdom; University of York, United Kingdom

## Abstract

Ongoing declines in production of the world's fisheries may have serious ecological and socioeconomic consequences. As a result, a number of international efforts have sought to improve management and prevent overexploitation, while helping to maintain biodiversity and a sustainable food supply. Although these initiatives have received broad acceptance, the extent to which corrective measures have been implemented and are effective remains largely unknown. We used a survey approach, validated with empirical data, and enquiries to over 13,000 fisheries experts (of which 1,188 responded) to assess the current effectiveness of fisheries management regimes worldwide; for each of those regimes, we also calculated the probable sustainability of reported catches to determine how management affects fisheries sustainability. Our survey shows that 7% of all coastal states undergo rigorous scientific assessment for the generation of management policies, 1.4% also have a participatory and transparent processes to convert scientific recommendations into policy, and 0.95% also provide for robust mechanisms to ensure the compliance with regulations; none is also free of the effects of excess fishing capacity, subsidies, or access to foreign fishing. A comparison of fisheries management attributes with the sustainability of reported fisheries catches indicated that the conversion of scientific advice into policy, through a participatory and transparent process, is at the core of achieving fisheries sustainability, regardless of other attributes of the fisheries. Our results illustrate the great vulnerability of the world's fisheries and the urgent need to meet well-identified guidelines for sustainable management; they also provide a baseline against which future changes can be quantified.

## Abstract 2

A global analysis shows that fishery management worldwide is lagging far behind international standards, and that the conversion of scientific advice into policy, through a participatory and transparent process, holds promise for achieving sustainable fisheries.

## Author Summary

Global fisheries are in crisis: marine fisheries provide 15% of the animal protein consumed by humans, yet 80% of the world's fish stocks are either fully exploited, overexploited or have collapsed. Several international initiatives have sought to improve the management of marine fisheries, hoping to reduce the deleterious ecological and socioeconomic consequence of the crisis. Unfortunately, the extent to which countries are improving their management and whether such intervention ensures the sustainability of the fisheries remain unknown. Here, we surveyed 1,188 fisheries experts from every coastal country in the world for information about the effectiveness with which fisheries are being managed, and related those results to an index of the probable sustainability of reported catches. We show that the management of fisheries worldwide is lagging far behind international guidelines recommended to minimize the effects of overexploitation. Only a handful of countries have a robust scientific basis for management recommendations, and transparent and participatory processes to convert those recommendations into policy while also ensuring compliance with regulations. Our study also shows that the conversion of scientific advice into policy, through a participatory and transparent process, is at the core of achieving fisheries sustainability, regardless of other attributes of the fisheries. These results illustrate the benefits of participatory, transparent, and science-based management while highlighting the great vulnerability of the world's fisheries services. The data for each country can be viewed at http://as01.ucis.dal.ca/ramweb/surveys/fishery_assessment.

## Introduction

Fisheries play an important role in the global provision of food, directly accounting for at least 15% of the animal protein consumed by humans and indirectly supporting food production by aquaculture and livestock industries [1],[2]. Demand for fish is expected to grow given escalating animal protein demands in developing countries and the rapidly increasing human population [1]–[4]. However, reported global marine fisheries landings have declined by about 0.7 million tonnes per year since the late 1980s [5], with at least 28% of the world's fish stocks overexploited or depleted, and 52% fully exploited by 2008 [1]. Severe reductions in abundance can change population genetic structure [6], harm the recovery potential of stocks [7], trigger broader ecosystem changes (e.g., [8]–[10]), threaten livelihoods [1], and endanger food security [11] and efforts towards the reduction of hunger [11],[12]. Given the different ecological and socioeconomic consequences of a global fisheries crisis, a number of international efforts have sought to improve management in the hope of moving towards sustainable marine fisheries (sensu Pauly et al. [13]). Some of these initiatives, which incorporated to varying degrees the improvement of marine fisheries management, include the United Nations Code of Conduct for Responsible Fisheries from the Food and Agriculture Organization [14], the Convention on Biological Diversity (http://www.cbd.int/), and the Millennium Ecosystem Assessment (http://www.millenniumassessment.org). Although these initiatives have received broad acceptance, the extent to which corrective measures are implemented and effective remains poorly known [15]–[17]. Using a survey approach, validated with empirical data and enquiries to fisheries experts, we quantified the status of fisheries management in each nation worldwide that has an exclusive economic zone (EEZ). We also related our measurements of management effectiveness to a recently developed index of fisheries sustainability. To our knowledge, these results represent the first global assessment of how fisheries management attributes influence sustainability, while providing a baseline against which future changes can be quantified.

## Results and Discussion

### Approach and Validation

**Figure 1:**
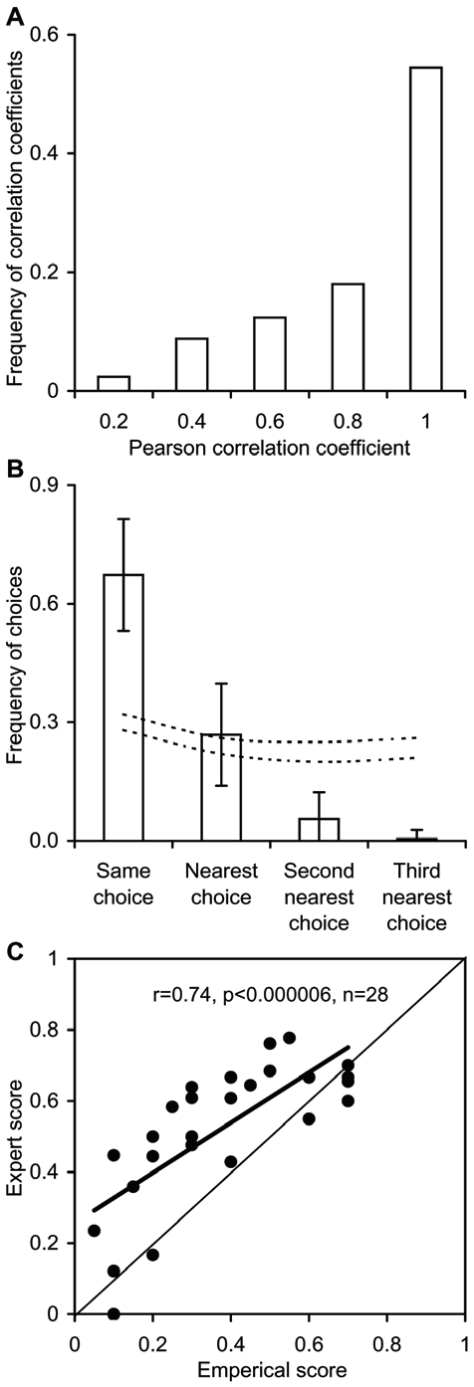
Reliability and validity of the expert's answers. Validity refers to the degree to which the responders' answers approach the truth. Reliability refers to the extent to which different experts agreed in their answers. (A) Using countries for which duplicated responses were obtained, we show the frequency distribution of the Pearson correlation coefficients contrasting each responder to other responders in the same country. (B) depicts the frequency with which responders chose the same score or the next closest choice. Dotted lines in the plot indicate the confidence limits of a null model in which the levels of agreement were measured when choices were made randomly. The confidence limits are based on 1,000 repetitions of this null model. The error bars indicate standard deviation. (C) Using empirical data collected by another study [15], we show the similarities between our expert-based score and an empirically based score for a particular question (see Materials and Methods). The diagonal line indicates the 1:1 ratio.

We evaluated the effectiveness of national fisheries management regimes by quantifying their degree of compliance with a well-recognized set of conditions necessary for sustainable fisheries: (1) robust scientific basis for management recommendations, (2) transparency in turning recommendations into policy, (3) capacity to enforce and ensure compliance with regulations, and minimizing the extent of (4) subsidies, (5) fishing overcapacity, and (6) foreign fishing in the form of fisheries agreements [8],[14]. The extent to which individual countries met or were affected by these conditions was quantified using a set of normative questions assembled in an Internet survey, which was systematically distributed to fisheries experts worldwide. Over 13,000 experts were contacted as part of this survey, of which 1,188 responded from each country bordering the ocean (i.e., EEZ; see Materials and Methods for additional details on areas surveyed). Experts were mostly fisheries managers, university professors, and governmental and nongovernmental researchers. Despite these diverse backgrounds, responses were highly consistent within each country (i.e., where multiple responses were given, 67% of experts chose the same answer to any given question and 27% chose the next closest response; Figure 1A and 1B) and in accordance with independent empirical data (we found a strong correlation between experts' opinions and empirical data [*r* = 0.74, *p*<0.00001, *n* = 28 countries; Figure 1C]). Justification, extended results, and discussion on the reliability and validity of the experts' data are presented in Materials and Methods. We also used a Monte Carlo simulation approach to include score uncertainty estimates in the results. We provide the main results and general conclusions in the text; full results are presented in Figures S1, S2, S3, S4, S5 and http://as01.ucis.dal.ca/ramweb/surveys/fishery_assessment/.

### Scientific Robustness

**Figure 2:**
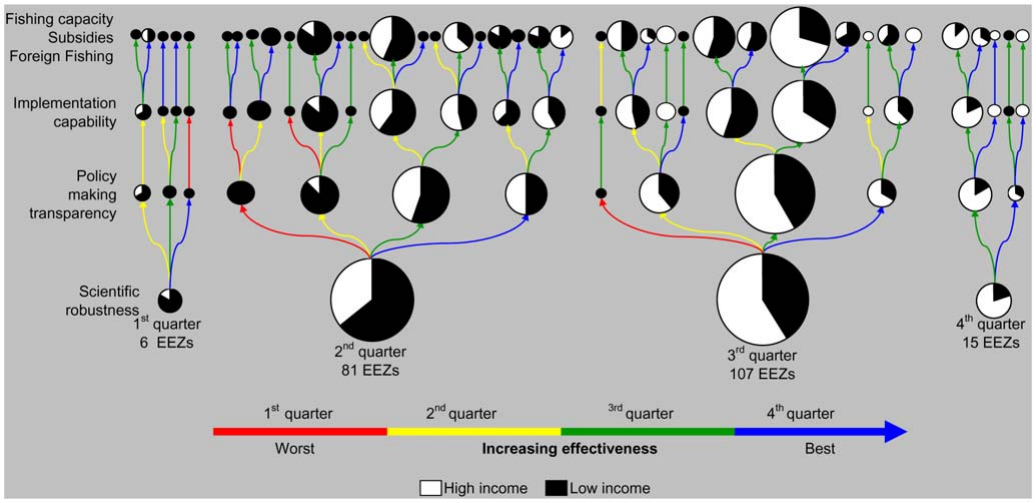
Discrimination of the world's exclusive economic zones (EEZs) according to their management effectiveness. Effectiveness is defined in terms of scientific robustness, policymaking transparency, implementation capability, and extent of fishing capacity, subsidies, and access to foreign fishing. Each attribute was quantified with a set of questions, whose answers were summarized into a single scale using multidimensional scaling (see Materials and Methods). For display purposes, each scale was divided into four quarters aligned from worst- to best-case scenarios (each quarter is color coded as indicated at the bottom of the figure). Our assessment of fishery management effectiveness started with the classification of all analyzed EEZs among the four quarters on the scale of scientific robustness. The EEZs within each of those quarters were then classified among the four quarters on the scale of policymaking transparency, and then those EEZs classified among the quarter of the next attribute, with the subdivision continuing until all EEZs were classified in all attributes. The size of the bubbles is proportional to the number of EEZs classified in each quarter. For purposes of display, subsidies, overcapacity, and fishery access agreements were summarized in a single scale with multidimensional scaling; full results are provided in the Figure S1.

**Figure 3:**
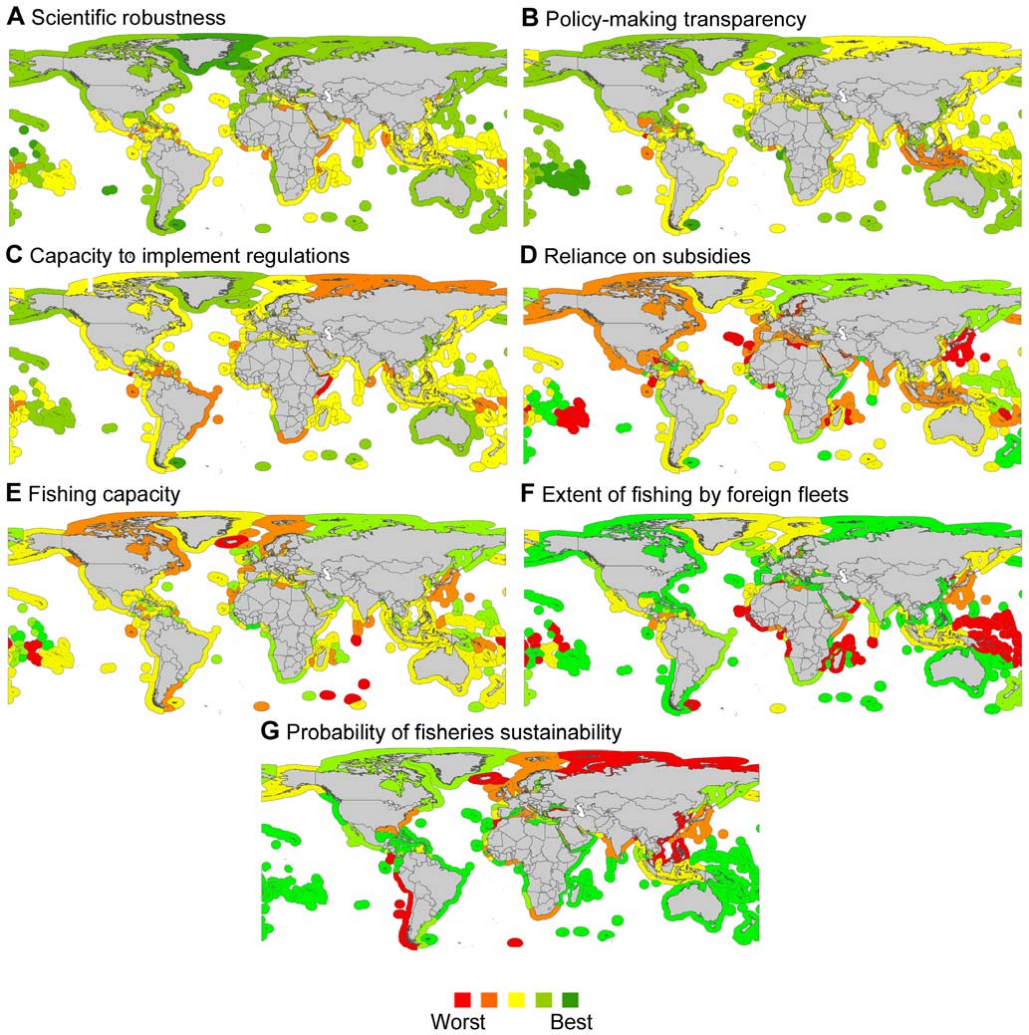
Management effectiveness and sustainability of the world's fisheries. These figures depict the results of experts' opinions on the valuation of scientific robustness (A), policymaking transparency (B), implementation capability (C), subsidies (D), fishing capacity (E) and access to foreign fishing (F). (G) depicts the probability that fisheries in each EEZ are sustainable (*P_sust_*) in 2004.

Critical to the success of fisheries management is the scientific basis on which management recommendations are made [18],[19]. Preventing the collapse of fisheries and ecosystem-wide impacts requires scientific advice in which uncertainty is minimized by using skilled personnel, models that include, not only the dynamics of fished stocks, but also their embedded ecosystems, and high-quality and up-to-date data (such that reliable recommendations can be adapted as conditions and stocks fluctuate). Alternatively, the effects of uncertainty can be minimized by applying precautionary approaches in the face of limited knowledge [18],[20]. Of the world's 209 EEZs analyzed, 87% have scientific personnel who are qualified (e.g., with Ph.D.- or Masters-level education, or have participated in training courses or relevant conferences) to perform fisheries assessments and provide science-based management advice (Figure S1A), approximately 7% use holistic models as the basis of management recommendations (i.e., including a broad set of biological and environmental data on fisheries to enable ecosystem-wide understanding of fisheries drivers and impacts; see Figure S1B), 61% carry out frequent assessments to ensure the effectiveness of existing management measures (Figure S1C), and 17% implement precautionary approaches for at least some species (Figure S1D). We summarized all responses that pertain to “scientific robustness” on a linear scale using multidimensional scaling. (Multidimensional scaling is an ordination method that uses the similarities and dissimilarities among responses to reduce the number of variables analyzed. This facilitates the assessment and visualization of patterns from several dimensions into one. Very simplistically, this is analogous to calculating an average of the different scores for each country; see Materials and Methods.) The resulting scale ranged from 0 to 1, and we divided it into four quarters (i.e., from 0 to 0.25, from 0.25 to 0.5, from 0.5 to 0.75, and from 0.75 to 1, with the lowest quarter indicating the worst combination of attributes and the top the best). We found that 7% of all EEZs rank in the top quarter of such a scale (Figure 2, countries depicted in Figure 3A), which account for approximately 9% of the world's fisheries catches and approximately 7% of the world's fished stocks (data are for 2004; see details in Figure S2). Distinguishing between high- and low-income countries using per capita Gross Domestic Product (i.e., 2007 per capita Gross Domestic Product larger or smaller than US$10,000, respectively), we found that high-income countries ranked significantly higher on the scale of scientific robustness (Mann-Whitney *U* test: *p*<0.00001, Figure S1E).

We note that a recent study indicated the success of catch shares, as individual transferable quotas, in preventing fisheries collapses [21]. This strategy has been implemented primarily in the EEZs of New Zealand, Australia, United States, Iceland, Chile, and Peru, which are all countries with robust scientific capabilities (Figure 3A). Our results indicate that the global adoption of individual transferable quotas should be considered with caution given that their underlying success rests on the scientific robustness of the implemented quotas and that few countries meet that condition (Figure 3A).

### Policy Transparency

Guidelines to improve the acceptance and compliance with fishing regulations recommend that decisions be based on the best available scientific evidence and follow a transparent and participatory process [8],[14],[22],[23]. Unfortunately, the process of policymaking can be subjected to substantial political pressures, perhaps including corruption. In our survey, management authorities from 92% of the EEZs consider scientific recommendations in formulating policies (Figure S1F), and in 87%, all stakeholders are consulted or their opinions considered (Figure S1G). Yet in 91% of all EEZs, regulations commonly face economic or political pressures to increase allowable catches or to implement regulations that err on the side of risk rather than caution (Figure S1I), whereas a surprising 83% of EEZs are thought to face corruption or bribery (Figure S1H). Of all EEZs, 26% rank in the top quarter of a scale of “policymaking transparency,” which summarizes, through multidimensional scaling, the attributes of considering scientific advice, participation, pressures, and corruption (Figure S1J, countries depicted in Figure 3B). Only 1.4% of all EEZs are in the top quarter on the combined scales of scientific robustness and policymaking transparency (Figure 2), which together accounted for 0.85% of the world's fisheries catch and 1.1% of the world's fished stocks (Figure S2). There were no significant differences between low- and high-income countries with respect to policy transparency (Figure S1J). However, the underlying mechanism was different, with low-income countries facing more corruption (*p*<0.00001, Figure S1H) and less commonly incorporating scientific advice (*p*<0.005, Figure S1F), whereas high-income countries faced slightly more political pressures (*p*<0.05, Figure S1I).

### Implementation Capability

One of the biggest challenges in fisheries management lies in the implementation and enforcement of regulations [23]. Poverty, unemployment, available infrastructure for control and surveillance, the severity of penalties for violations, and participation in policymaking are all likely influencing the level of compliance with regulations. Proper enforcement through (1) adequate funding and equipment for the managing authorities, (2) patrolling of fishing grounds, and (3) tough penalties for infringements, occurs in 17% of all EEZs (Figure S1K; note that only ∼6% of all EEZs impose penalties that are sufficiently tough to deter violators). Not surprisingly, no EEZ was free of the effects of poaching (Figure S1L, see also [24]). On a scale of “implementation capability,” which summarizes, through multidimensional scaling, poaching and the different attributes of enforcement, we found that only approximately 5% of all EEZs are in the top quarter of such a scale (Figure S1M, countries depicted in Figure 3C). Only two relatively small EEZs, those of the Faeroe and Falkland Islands, were in the top quarter for all three indicators of scientific robustness, policymaking transparency, and implementation capability (Figure 2), which combined, accounted for 0.80% of the world's fisheries catch and 0.48% of the world's fished stocks (Figure S2). Better “implementation capability” is frequently more common among high- than low-income countries (*p*<0.0001, Figure S1M), which is mainly a consequence of better enforcement (*p*<0.00001, Figure S1K) and reduced poaching in the former (*p*<0.002, Figure S1L).

### Extent of Subsidies, Overcapacity, and Foreign Fishing

When the structure of a management regime is weak, fisheries will be prone to overexploitation due to several factors. Three that have received particular attention are fishing capacity, subsidies, and access to foreign fishing fleets [8],[23],[25],[26]. Open access to fishing (because of lack of effective management) leads to a “race for fish” that commonly increases fleet size and fishing power. This should reduce fish stocks, at which point fishing capacity should stabilize given decreasing profits from reduced catches [8]. Subsidies can override this mechanism by keeping fisheries profitable and encouraging overexploitation [8],[13]. The picture is further complicated by fisheries agreements that allow foreign fleets to catch fish that are not caught by national fleets [25],[26]. Unfortunately, such agreements are commonly made between developing coastal and island states (often with low capacity to assess stocks and to enforce regulations) and developed and heavily subsidized nations [25]. Recent analyses of current agreements indicate a high risk of overexploitation due to several reasons, including selling fishing rights on highly migratory stocks under bilateral agreements, selling access rights without specified catch limits, excessive by-catch, and distortion of reported catches, among others [25],[26]. Such agreements are thought to develop coastal economies through monetary gains and local employment. In certain instances, revenues are also used to generate management plans; their effectiveness, however, is unclear given chronic weaknesses in fisheries governance and management systems [25].

Our assessment of the extent of fishing capacity, subsidies, and access to foreign fishing fleets yielded the following results. We found that fleet sizes are quantified and regulated in 20% of the world's EEZs (Figure S1N), although in 93% of EEZs, fishing fleets face some level of modernization to catch fish more efficiently or cheaply (Figure S1O). Thus, although fishing capacity may be reduced in terms of fleet size, fishing power may remain constant or even increase due to technological improvements (i.e., fewer improved boats being more effective at catching fish). Effective controls on fleet size were more common among high-income than low-income EEZs (*p*<0.02, Figure S1N), but the former modernized their fleets more often than the latter (*p*<0.00001, Figure S1O). Using multidimensional scaling to summarize the results pertaining to “fishing capacity” (i.e., fleet size controls and fleet modernization), we found high-income EEZs having significantly higher fishing capacity than low-income ones (*p*<0.02, Figure S1P, countries depicted in Figure 3E). Fisheries sectors that rely to some degree on subsidies occurred in 91% of the world's EEZs (Figure S1Q; countries depicted in Figure 3D), and more commonly among high- than low-income EEZs (*p*<0.00001, Figure S1Q) (see also [27]). Access to foreign fishing is granted in 51% of all EEZs (Figure S1R, countries depicted in Figure 3F), and is more frequent in low- than high-income EEZs (*p*<0.00001, Figure S1R). In fact, our survey indicated that in 33% of the EEZs that are classified as low income (commonly, countries in Africa and Oceania), most fishing is carried out by foreign fleets from either the European Union, South Korea, Japan, China, Taiwan, or the United States (Figure S3). No single EEZ meets the best standards (i.e., top quarter of the scales) of scientific robustness, policymaking transparency, and implementation capability while being free of the effects of excess fishing capacity, subsidies, or access to foreign fishing (Figure 2).

### Extent and Management Control of Recreational and Small-Scale Fisheries

The notion that industrialized fishing practices are solely responsible for the global fisheries crisis has been challenged by evidence of the significant effects of recreational and small-scale commercial or subsistence fisheries (e.g., [28],[29]). Although less intensive per unit area, small-scale and recreational fisheries can be far more extensive spatially. Small-scale and recreational fisheries are important in 93% and 76% of the world's EEZs, respectively (Figure S4), and small-scale fisheries are increasingly more predominant among low-income EEZs whereas recreational fisheries are more predominant in high-income countries (*p*<0.0001, Figure S4). Of the world's EEZs, 40% collect at least some data on small-scale fishing, and 13% on recreational fishing; 30% impose regulations on the size of fish caught in small-scale fishing, and 29% do so for recreational fishing, 7% regulate the number of fish caught in small-scale fishing, and 15% do so for recreational fishing, whereas 10% limit the number of fishers in small-scale fisheries, and 3% do so for recreational fishing (Figure S4). These management measures are more frequent in high- than low-income EEZs (Figure S4). Measures to regulate small-scale and recreational fishing are clearly limited and could prove detrimental to food supply and sustainability if they continue to operate outside the control of fisheries management institutions.

### Overall Management Effectiveness

**Figure 4:**
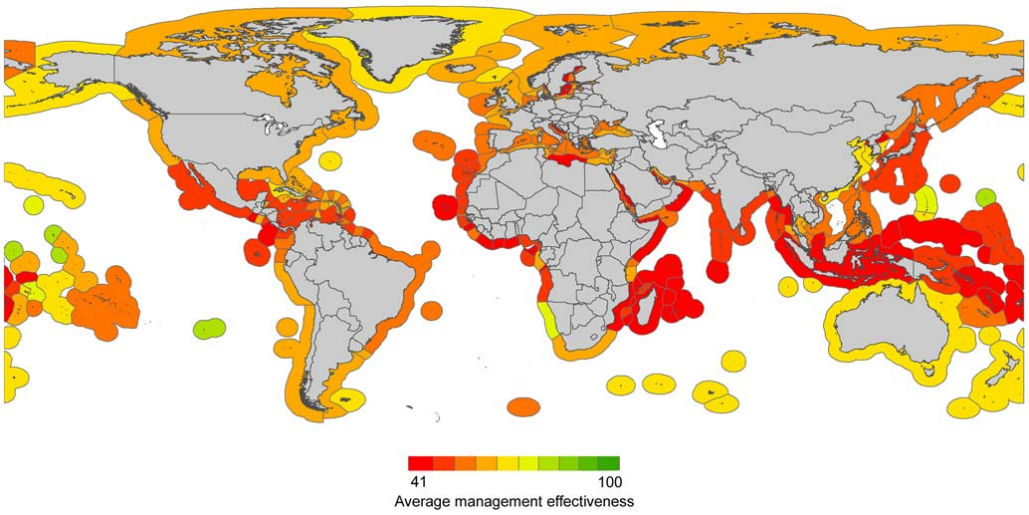
Overall management effectiveness of the world's exclusive economic zones. This map shows the average, for each surveyed area, of their scores on the scales of scientific robustness, policymaking transparency, implementation capability, fishing capacity, subsidies, and access to foreign fishing.

To provide a general overview of fisheries management effectiveness, we averaged all scores on the scales of scientific robustness, policymaking transparency, implementation capability, fishing capacity, subsidies, and access to foreign fishing. We excluded the effects of small-scale and recreational fisheries, recognizing that their lack of management would extensively reduce the scores. Only 5% of all EEZs were in the top quarter of this scale (Figure S1S, countries depicted in Figure 4), with high-income EEZs having significantly better overall management effectiveness than low-income ones (*p*<0.00001, Figure S1S). A sensitivity analysis indicated that the difference between high- and low-income EEZs was driven mainly by foreign fishing agreements, which disproportionally reduced the average score of low-income EEZs. Excluding foreign fishing access leads to similarly low average scores between high- and low-income EEZs (Figure S1S). Similar average scores are, however, explained by different mechanisms, namely excessive fishing capacity and subsidies in high-income EEZs and deficient scientific, political, and enforcement capacity in low-income EEZs (Figure S1).

### Effect of Fishery Management on Fisheries Sustainability

One final question that we addressed in this study is to what extent the different attributes of fisheries management analyzed here relate to the actual sustainability of fisheries. We addressed this question using a recently developed method to quantify the probability that ecosystems are being sustainably fished (*P*_sust_). This metric assesses the probability that the ratio between the biomass losses due to fishing (i.e., total catch) expressed in primary production equivalents and the primary production of the area in which the catch was taken is sustainable (see Materials and Methods, [30],[31]). We found that this metric is particularly useful to differentiate misinterpretations in landings data when used as an indicator of fisheries status (Figure S5). The metric, for instance, differentiates between countries in which increasing landings (a possible symptom of good fisheries status) are sustainable or not, and between countries in which declining landings (a possible symptom of overfishing or enhanced management [32]) are indicative of the sustainability of fisheries or not (Figure S5). We used classification/regression tree analysis to identify the most likely management attributes that affect the probability of fisheries sustainability; we also included country wealth (i.e., the distinction between high and low income) in the classification tree to analyze differences in fisheries sustainability due to this factor.

**Figure 5:**
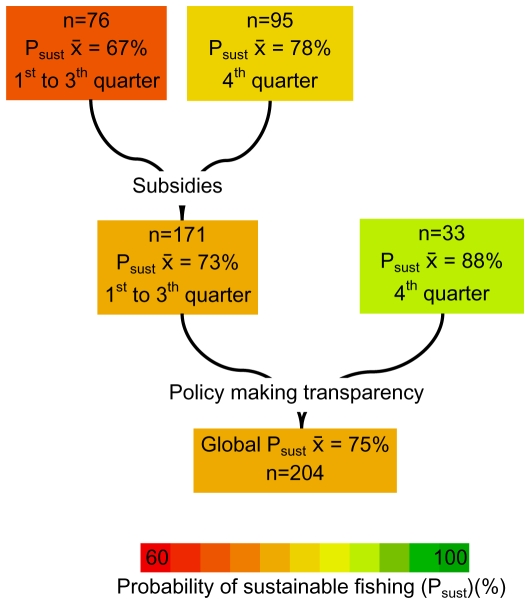
Effect of fishery management on fisheries sustainability. Results of a classification tree aimed to identify the most likely fishery management attributes related to the sustainability of fisheries. In a classification/regression tree, the factor that maximizes differences in fisheries sustainability is placed at the root of the tree, and the EEZs in each of its quarters are separated into different branches. This method repeatedly tests for significant differences among the EEZs in each branch in the remaining attributes and stops when no significant difference exists in any attribute within the EEZs of any branch (see Materials and Methods). The results shown here include the linking between the probability of fisheries sustainability (*P*_sust_) and each of the management attributes analyzed: scientific robustness, policymaking transparency, implementation capability, fishing capacity, subsidies, access to foreign fishing, and country wealth.

Of all management attributes analyzed (i.e., scientific robustness, policymaking transparency, implementation capability, fishing capacity, subsidies, and access to foreign fishing) plus taking into account country wealth, we found that variations in policymaking transparency led to the largest difference in fisheries sustainability. We found that EEZs ranked in the upper best quarter on the scale of transparent policymaking (i.e., EEZs where scientific advice is considered and followed, all parties are consulted and considered, and where corruption and external economic and political pressures are minimal [see Figure S1F–S1I]) show the largest probability of having sustainable fisheries compared to EEZs ranked in any of the other three quarters (Figure 5). The probability of sustainability in policy transparent EEZs was 88% compared to 73% in others (Figure 5). We also found that subsidies have an additional negative effect on fisheries sustainability among EEZs with nontransparent policy systems. We found that the probability of fisheries sustainability in nontransparent EEZs was reduced from 78% to 67% due to the effects of even modest subsidies (Figure 5) (i.e., EEZs ranked in the first three quarters on the scale of subsidies or EEZs in which fisheries sectors are dependent minimally to almost entirely on subsidies).

The significant effect of policymaking transparency on fisheries sustainability likely relates to the fact that this particular attribute forms the core of the fisheries management process. Firstly, it determines the extent to which scientific advice will be translated into policy, whereas transparent and legitimate participation of involved parties is likely to promote compliance with regulations [22]. Our findings indicate that policymaking transparency is likely to work as a “sustainability bottleneck” through which other positive attributes of fisheries management are filtered. For instance, we found that scientific robustness did not influence the sustainability of fisheries. This may be because, in the process of policymaking, scientific advice may be overridden due to socioeconomic costs and political or corruption pressures. The recent catch quotas for Mediterranean Bluefin tuna (*Thunnus thynnus*) established by the International Commission for the Conservation of Atlantic Tunas may serve as an example. In this particular case, robust and well-founded scientific advice recommended to maintain catches at 15,000 tonnes per year and to close the fisheries during two spawning months; yet the policy was set at 22,000 tonnes per year, with fishing allowed during critical spawning months. This is a case in which scientific robustness may not necessarily result in sustainability due to significant pressures in the process of policymaking. We also found that variation in implementation capabilities did not have much effect on fisheries sustainability. This result can also be explained by the effect of policymaking transparency. If the policymaking process is participatory and legitimate, it is likely that even poorly enforced systems will move towards sustainability because of voluntary compliance [22]. In contrast, some systems may strongly enforce regulations, but if the regulations were flawed during the process of policymaking, good enforcement may not bring about sustainability either. If the establishment of regulations includes scientific advice and follows a participatory mechanism, it is likely that fisheries will be tightly regulated, regardless of who carries out the fishing, which may also explain the lack of significance of fishing capacity and international fisheries agreements on fisheries sustainability. This is not to say that fishing capacity and foreign fishing access do not have impacts on fisheries sustainability but rather that their effects are moderated by the policymaking process (i.e., fishing capacity and access agreements may have different effects on sustainability in situations that are tightly regulated compared to those that are not). Finally, our results indicate how deficiencies in the process of policymaking can leave fisheries vulnerable to overexploitation due to the effect of subsidies. It is known that subsidies can override possible fishing controls exerted by economic benefits (see section above on subsidies; [8],[13],[27]). We presume, however, that this effect is likely to be more pervasive in nontransparent systems given that fishing remains poorly controlled or regulated and allowed to fluctuate more freely, depending largely on subsidies.

### Concluding Remarks

Improvements to fisheries management have been incorporated into international initiatives, which have received broad acceptance (e.g., [14],[15]). Unfortunately, our study shows that there is a marked difference between the endorsement of such initiatives and the actual implementation of corrective measures. The ongoing decline in marine fisheries catches [5],[9],[33]–[36] and the ecological and socioeconomic consequences of a fisheries crisis call for a greater political will of countries worldwide if further fisheries declines and their wider consequences are to be prevented. Effective transfer of improved scientific capacities to policy, achieved through a transparent and participatory process, will be more important than ever in stabilizing our food supply from the sea and preventing unnecessary losses due to management deficiencies. Current projections suggest that total demand for fisheries products is likely to increase by approximately 35 million metric tonnes by 2030 (∼43% of the maximum reported catch in the late 1980s) [3],[4] and by approximately 73% for small-scale fisheries by 2025 [35]. This contrasts sharply with the 20% to 50% reduction in current fishing effort suggested for achieving sustainability [30],[36], and implies that regulators may face increasing pressures towards unsustainable catch quotas. Given that the demand for fish lies outside the control of conventional fisheries management, other national and international institutions will have to be involved to deal with poverty alleviation (inherently improving management, Figure S1) and stabilization of the world's human population (to soften fisheries demand), if pressures on management are to be prevented and sustainability achieved.

## Materials and Methods

### Conditions Analyzed

We considered factors broadly recognized as critical for the sustainable management of fish stocks (by sustainability, we mean sustainable catches and not social, economic, or institutional sustainability and the like, which at times are also associated with fisheries management and often dominate policy decisions). The factors considered in the present analysis were categorized into those related to the robustness of scientific recommendations, transparency in the process of converting recommendations into actual policy, the capability to enforce and ensure compliance with regulations, and the extent of fishing capacity, subsidies, and access to foreign fishing. Each of these attributes was evaluated with a set of questions whose answers could be categorized in a hierarchical order from worst- to best-case scenarios. In cases where several questions applied to the same attribute, we summarized all responses into a single scale using multidimensional scaling. Multidimensional scaling is an ordination method that uses similarities and dissimilarities among variables to reduce them to a specific number of dimensions. Here, we used the anchored multidimensional scaling method developed by Pitcher and Preikshot [37]. In this method, hypothetical countries are generated with the worst- and best-case scenarios for each question and used as normative extremes of a scale on which real countries are ranked. The approach also incorporates uncertainty using a Monte Carlo simulation tool based on the maximum and minimum possible for each score [38]. A copy of the software is available on request.

### Fishery Management Regimes Analyzed

We focused our assessment on fishery management conditions for all ocean realms under the sovereignty of a defined coastal territory. Under the United Nations Convention on the Law of the Sea [39], the protection and harvesting of coastal resources rest within the 200-nautical mile EEZ of each coastal state. There are, however, exceptions, such as the European Union, whose fisheries regulations are mandated by the Common Fisheries Policy but whose enforcement is the responsibility of the member states; member states also differ in their fishing capability and possibly in their compliance with regulations. Similarly, many countries have overseas territories, which may or may not have autonomous control of the regulation of their fisheries, and consequently, there may be variations in the effectiveness of their management regimes. For instance, Saint Pierre and Miquelon, French Guiana, French Polynesia, French Southern and Antarctic lands, New Caledonia, Saint Martin, Reunion, Guadeloupe, and Martinique all are under the sovereignty of France, which furthermore has direct control over its own Atlantic and Mediterranean coast; yet all of these zones have different management conditions. To consider these differences in fishery management regimes, zones managed under the same entity (e.g., the European Union) or zones in different parts of the world belonging to the same sovereignty (e.g., overseas territories of France, United Kingdom, and United States) were analyzed separately. We also included zones that may not be technically defined or recognized as EEZs under the United Nations (e.g., division among coastal states of the Baltic Sea and Black Sea). In total, 245 such zones exist in the world (see Figure 3), which excludes conflict zones (e.g., the Paracel Islands, Spratly Islands, and Southern Kuriles). Out of those 245 zones, we were unable to gather data for isolated islands under the sovereignty of the United Kingdom (i.e., Ascension, Pitcairn, Saint Helena, South Georgia, and the South Sandwich Islands and Tristan da Cunha) and France (Clipperton Atoll) for which neither contacts nor information was available. We also excluded Monaco and Singapore; interviewees at local authorities (Coopération Internationale pour l'Environnement et de Développement in Monaco and the Agri-Food and Veterinary Authority in Singapore) in both of these countries claimed that although marine fishing occurs, it was minimal and considered insufficient to motivate governmental regulation. The final database contained complete data for 236 zones. Although all data are reported in Figures 3 and 4, the statistics reported in the text were based on 209 inhabited zones for which per capita Gross Domestic Product data exist; that excluded uninhabited and isolated atolls to prevent biases due to the fact that we could not get data for all such areas (i.e., United Kingdom and France, see above).

### The Survey

For each of the attributes analyzed (i.e., scientific robustness, policymaking transparency, enforcement capability, fishing effort control, subsidies, and access to foreign fishing), we created a set of questions whose answers could be ranked on a scale from worst- to best-case scenarios. The resulting survey included 23 multiple choice questions and was posted on the Internet (http://as01.ucis.dal.ca/ramweb/surveys/fishery_assessment/) in five different languages (i.e., English, Spanish, French, Portuguese, and German). We searched for contacts (email addresses and phone numbers) of fishery experts for all coastal territories in the world. Our sources of information were reports on scientific and administrative meetings relevant to fisheries, Web pages of nongovernmental organizations, Web pages of fishery management organizations in each territory, and proceedings of international conferences on fisheries. The final directory included contact information for 13,892 people. We sent personalized emails using recommendations of email marketing companies to prevent filtering of emails by local servers and promote participation. The survey started in April 2007 and was completed in April 2008. For zones where we did not receive an email response, we carried out phone interviews with local experts, and both email and phone queries were done until at least one full set of responses was available for each zone. We received 1,188 positive responses including at least one from each country with ocean access. Multiple responses for the same zone were averaged.

### Justification of the Approach and Assessment of Responders' Reliability and Validity

Expert opinion surveys have been very popular in social, medical, political, and economic sciences [40], and some examples exist in fisheries studies (e.g., [41]). In fisheries research, expert opinions have been categorized as a “highly reliable” method given that overall, it works as a form of “peer review approach” and, for some crucial issues, is the only knowledge available (see [42]). The approach is also cost-efficient and relatively fast. The collection of empirical data for an analysis of this scale could prove ineffective because country-scale data are patchy, in most cases inaccessible through traditional searching engines, and because old data may not describe current conditions. For these reasons, we chose the survey of local experts to acquire data.

The quality of expert opinion surveys relies on the consistency of responders and their understanding of the issues. These problems are defined as reliability and validity [40], which in statistical terms are analogous to precision and accuracy. The former basically considers the extent to which responders agree in their responses and the latter the extent to which the responses approach the truth. Evaluation of data reliability and validity also allows assessment of the extent of expert biases, which may arise for different reasons (e.g., cultural differences, patriotism, opposition to governmental institutions, etc.). Our assessment of reliability and validity was as follows:

#### Reliability

To test the extent of consistency among responders, we used data from EEZs for which duplicated responses were received. We performed individual Pearson correlations between each responder and the group of responders (recommended by Fleiss [40]). We also tested the significance of the levels of agreement by comparing the actual levels of agreement among responders with the levels of agreement expected when choices were made randomly (see Figure 1). Analyzing 259 independent responses for 17 EEZs, we found a high level of agreement among responders, with over 72% of the cases showing Pearson correlation coefficients greater than 0.8 (Figure 1A). This was due to the fact that in 67% of the cases, the responders chose exactly the same score for any given question, and in 27%, the nearest choice (Figure 1B). Only in 5% of the cases did the responders differ by more than one choice, and in 0.4%, they chose opposite scores (Figure 1B). The levels of agreement and disagreement were significantly higher and lower, respectively, than those expected by chance (Figure 1B). These high levels of agreement are very likely due to the fact that questions were general and the possible responses relatively broad. Under these conditions, responses by different responders are most likely to converge on similar or closely related scores.

#### Validity

The survey allowed questions to be left unanswered so that responders could answer only the questions they knew about. Most commonly, responders voluntarily, and at times upon our request, gave contact information for other people better placed to provide missing answers. To address the issue of validity, our survey included a question on the extent to which countries are rebuilding depleted fish stocks, an issue explicitly covered by The United Nations Code of Conduct (Article 7, clause 7.6.10), and evaluated in a survey carried out by Pitcher et al. [15]. The scores from the two different sources (i.e., expert-based and empirically based) for the countries in common were rescaled from 0 to 1 for comparison, and similarities evaluated using a Pearson correlation. This analysis was based on 28 countries for which empirical data were available and reliable to assign an empirical score. The results of this analysis indicated a strong correlation between expert opinion and empirical data (*r* = 0.74, *p*<0.000006, Figure 1C), although expert opinion tended to overestimate the extent to which countries are rebuilding their depleted fisheries (Figure 1C). Thus, the overall statistics provided here should likely be considered a conservative (more optimistic) view of the actual situation.

### Quantification of Fisheries Sustainability

The metric we used to quantify fisheries sustainability has been recently published in two independent publications [30],[31], but not applied to the landings of any country. Here, we provide a brief description of its rationale and calculation, but extended details are provided by Libralato et al. [31] and Coll et al. [30].

Fisheries catches represent a net export of mass and energy that can no longer be used within an ecosystem; failure of the ecosystem to compensate for that energy loss implies overexploitation. This notion of overexploitation will require establishing a contrast between the loss of energy in the ecosystem due to a particular catch, the energy at the base of the food web in the area where the catch was taken, and reference points indicating whether the ratio between the energy that is taken (by fishing) and produced (through primary production) is sustainable or not. This concept has been recently incorporated into a metric that aims to quantify the probability that an ecosystem is being sustainably fished (*P*_sust_: after [31]). The metric first calculates the amount of Primary Productivity Required (*PPR* after [43]) to sustain a catch as 
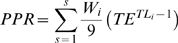
, where *s* is the total number of caught species, *W_i_* is catch weight of each species *i*, *TE* is transfer efficiency specific for the ecosystem, and *TL_i_* is the trophic level of species *i*. The metric assumes a conservative 9:1 ratio for the conversion of total weight to carbon [43]. The loss of energy in the ecosystem (i.e., *L*_index_, after [31]) is calculated by comparing PPR to the primary production at the base of the food web (*PP*) as 
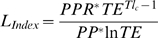
, where *TL_c_* is the mean trophic level of the catch as calculated from the TL and weight of each species in the catch. *PP* is parameterized from chlorophyll pigment concentrations and photosynthetically active radiation [30]. The probability that such energy loss is sustainable (i.e., *P*_sust_) is calculated by comparing *L*_index_ to reference *L*_indexes_ in which overfishing or sustainability have previously been identified. Reference *L*_indexes_ were quantified for different regions worldwide using a set of well-documented mass balance models representative of exploited ecosystems and constructed with independent information for each ecosystem [31]. Each of these models is classified as overfished if it meets one or more of the following criteria: (1) biomass of any species falls below minimum biologically acceptable limit, (2) diversity decreases, (3) year-to-year variation in populations or catches increases, (4) resilience to perturbations decreases, (5) economic and social benefits decrease, and (5) nontargeted species get impaired (see [30],[31] and references therein for justification of these criterion). Models were defined as sustainable when the impacts of exploitation did not result in any of the above symptoms. The frequency of sustainable or overfished *L*_indexes_ allowed us to calculate the probability of sustainability (*P*_sust_) for any particular *L*_index_ value as 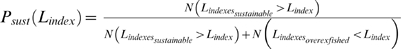, where *N* is the number of models in which *L*_indexes_ lead to sustainable or overfishing conditions. Probabilities of fisheries sustainability were calculated for each EEZ in the world using catch data as from the Sea Around Us fisheries database, which contains harmonized data from a variety of sources including the Food and Agriculture Organization (i.e., statistics on fisheries catches from 1950 to 2004; [44]). That database adjusted landings data to account for the fishing of long-distance fishing fleets (i.e., landings that are reported by one country, but fished in a different one). Landings data were also adjusted to include discards [45] and a global estimate of illegal, unreported, or unregulated catches [46],[47].

### Linkage between Management Effectiveness and Fisheries Sustainability

Data on fisheries sustainability was quantified for the year 2004 and linked to the effectiveness of fisheries management using a classification/regression tree. A classification tree tests for significant differences in fisheries sustainability among the quarters of each attribute (note that the first and fourth quarters are the extremes of a scale from worst- to best-case scenarios for each attribute; see Figure 2). The attribute that maximizes differences among quarters (i.e., smallest *p*-value) is placed at the root of the tree and the EEZs in each of those quarters separated in different branches. Subsequently, the EEZs in each branch are tested for significant differences among quarters of the remaining attributes. The attribute that maximizes differences among quarters is placed at the base of the branch and the EEZs in each of those quarters separated in upper branches. The process is repeated until no differences are found within each branch in any remaining attribute. This analysis included all attributes considered in this study: scientific robustness, policymaking transparency, implementation capability, fishing capacity, subsidies, access to foreign fishing, and country wealth (i.e., 2007 per capita Gross Domestic Product larger or smaller than US$10,000, respectively). Given the inflation of Type I errors due to multiple comparisons, significance was set at *p*<0.01.

## Supporting Information

Figure S1 Variations in the number of countries with different qualities in their fishery management attributes.
(0.48 MB PDF)

Figure S2 Discrimination of the world's fisheries catch and fished stocks according to different fishery management attributes.
(0.84 MB TIF)

Figure S3 Countries with the largest use of foreign fishing access agreements.
(9.31 MB TIF)

Figure S4 Global extent of recreational and small-scale fisheries and the frequency of countries imposing different types of regulations.
(0.50 MB TIF)

Figure S5 Robustness of the metric used to assess fisheries sustainability.
(7.05 MB TIF)

Text S1 Extended acknowledgements of the participants.
(0.04 MB DOC)

## Abbreviations

EEZ: exclusive economic zone
*L*_index_: loss of energy in the ecosystem
*P*_sust_: probability of sustainability of reported catches
