# Supplementary material for: Management Effectiveness of the World's Marine Fisheries
Source: PLoS Biol. 2009 Jun 23;7(6):e1000131. doi: 10.1371/journal.pbio.1000131 (PMC2690453; doi:10.1371/journal.pbio.1000131)

Figure S1. Variations in the number of countries with different qualities in their fishery management attributes. Charts on the left indicate the frequency distribution of countries in each attribute whereas those on the right distinguish between high- (grey lines) and low-income (black lines) countries. To test for significant differences in the frequency distribution between high- and low-income countries we used Mann-Whitney U tests and controlled for Type I-errors arising from multiple comparisons using the sequential Bonferroni-test [51]. In theory, one out of 20 contrasts may be statistically significant by chance alone [51-52], in our case the use of the sequential Bonferroni-test removed the significance of four comparisons out of 20 (indicated with * besides the p-values in the charts), which indicates the sometimes conservative nature of this test and inflation of Type II-errors [e.g. 52]. To control for this problem, we complemented the analysis with a null model in which the frequency distributions of high- and low-income countries were compared against the frequency distribution resulting from an equal number of countries selected randomly from the pool of available countries. Dotted lines on the right-hand charts indicate the confidence limits of repeating that model 1000 times. The approach is useful to determine where the frequencies actually differ from what is expected to occur by chance. Plot **A** was based on the responses to question # 4 of the survey, plot **B** on the answers to question # 2, plot **C** on the answers to question # 3, and plot **D** on the answers to question # 5. Plot **E** is the concatenation of the first four plots using multidimensional scaling (See methods in supplementary materials). Plot **D** includes data on the proportion of fisheries whose regulations are based on data from landings, by-catch and discards, mortality (as landing plus by-catch and discards), population size, recruitment, age structure, fish movements, environmental variables and ecological linkages.

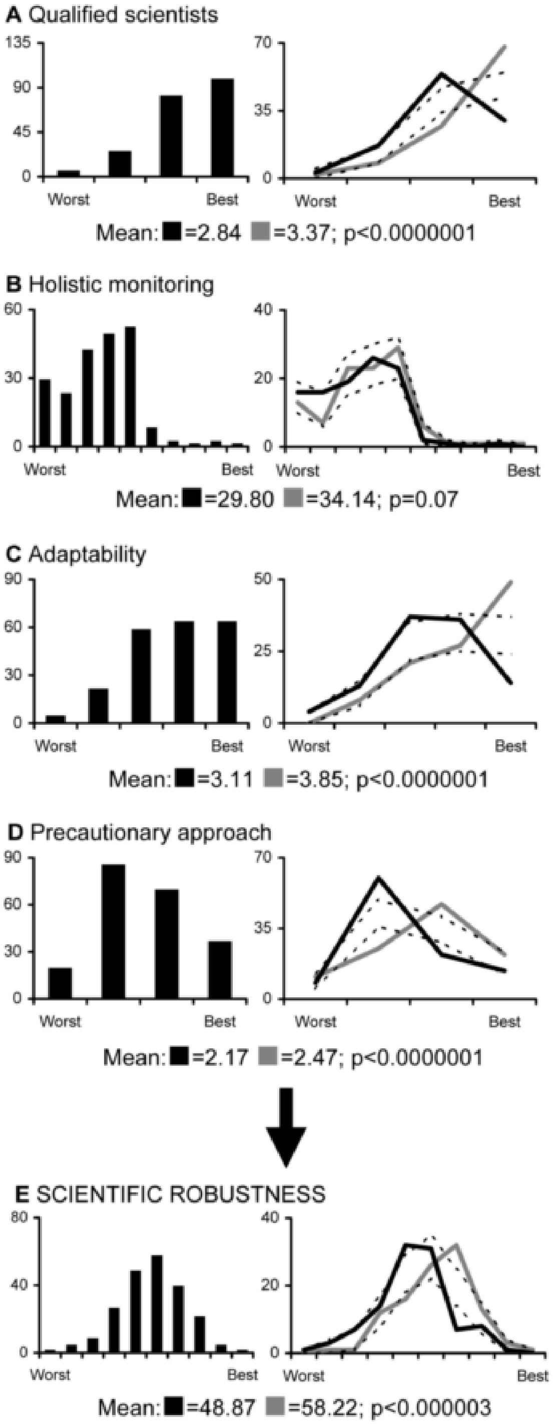

Cont. Figure S1. Responses used for the quantification of “Policy-making transparency”. Plot **F** was based on the responses to question # 13 of the survey, plot **G** on the answers to question # 11, plot **H** on the answers to question # 10 and plot I on the answers to question # 12. Plot **J** is the concatenation of the upper plots using multidimensional scaling.

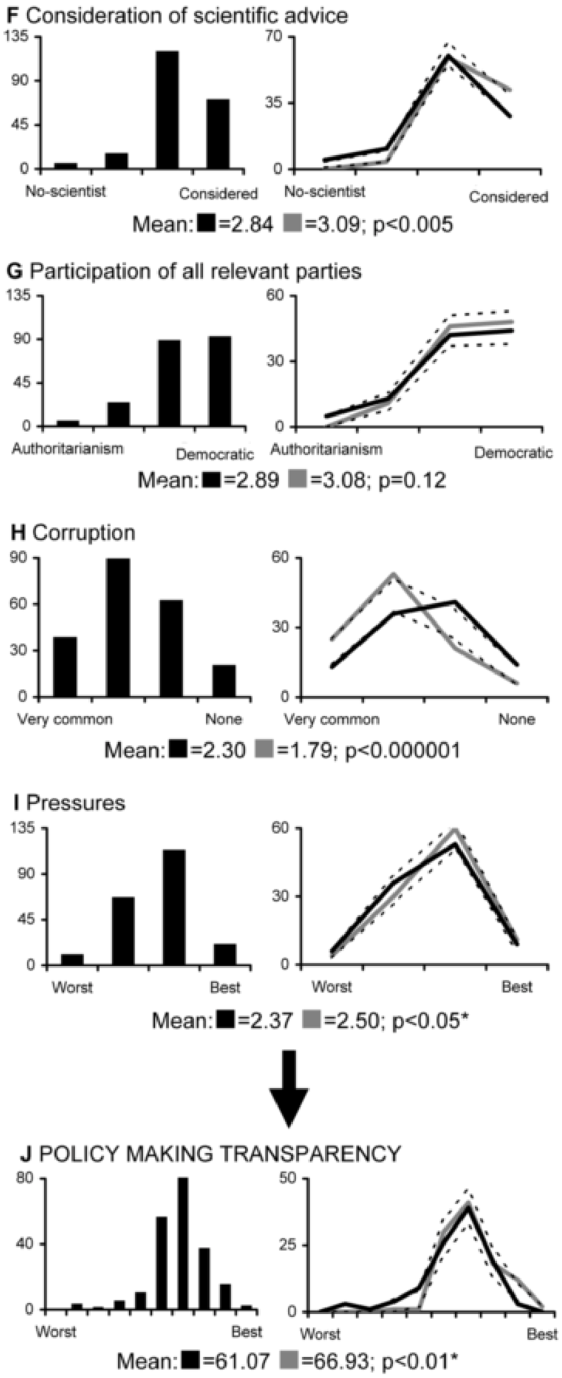

Cont. Figure S1. Responses used for the quantification of “implementation capability”. Plot **K** concatenated data on *i*) funding and equipment for the fishing authority to monitor patrol and enforce regulations [i.e. question #6], *ii*) frequency of patrols [i.e. question #7], and *iii*) penalties upon violators [i.e. question #9]. Plot **L** was based on the responses to question # 8 of the survey. Plot **M** is the concatenation of the upper two plots using multidimensional scaling.

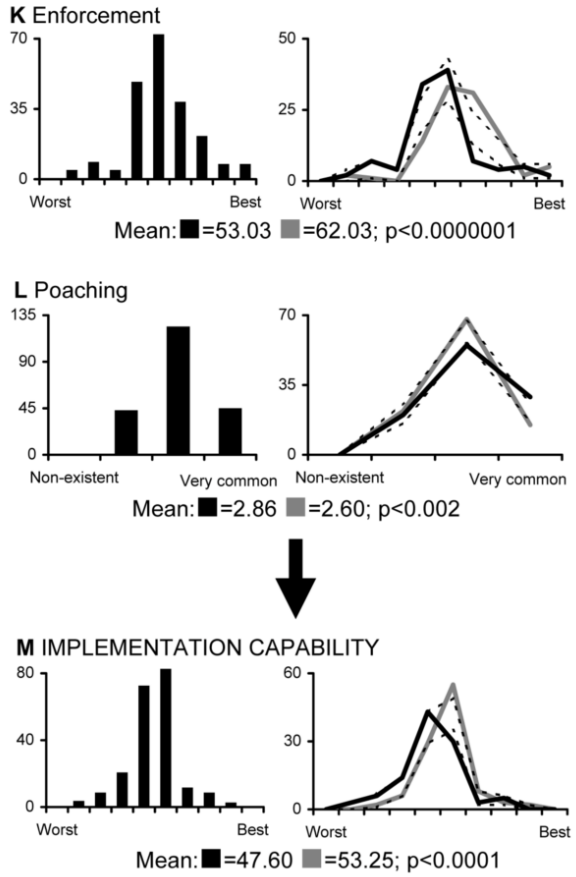

Cont. Figure S1. Responses used for the quantification of "fishing capacity". Plot **N** was based on the responses to question # 14 of the survey and plot **O** on the answers to question # 15. Plot **P** is the concatenation of the upper plots using multidimensional scaling.

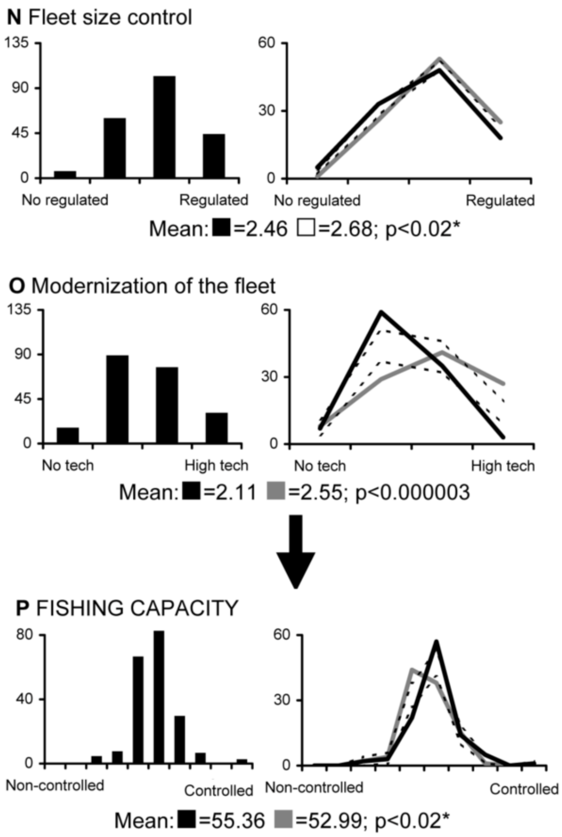

Cont. Figure S1. Responses to the attributes on subsidies and access to foreign fishing. Plot **Q** was based on the responses to question # 20 of the survey and plot **R** on the answers to question # 21.

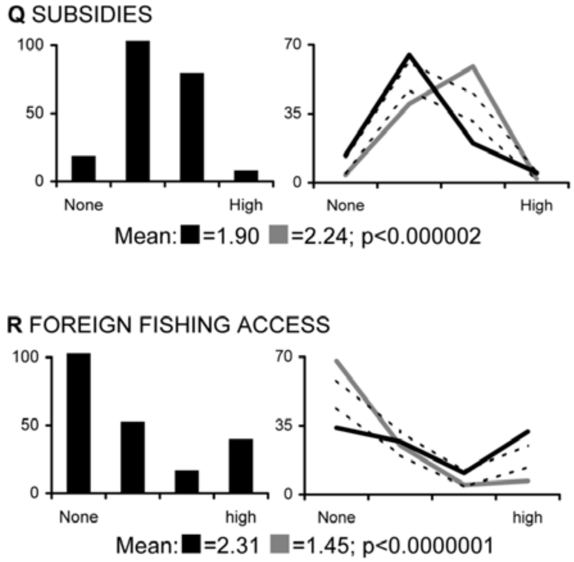

Cont. Figure S1. Averaging of attributes to calculate the average achievability of sustainability. Upper plot **S** averages the scores on scientific robustness, policy-making transparency, implementation capability, overcapacity, subsidies and foreign fishing access. A sensitivity analysis indicated that while average scores were significantly better among high- than low-income EEZs, this difference was driven mainly by foreign fishing agreements, which disproportionally reduced the average score of low-income EEZs. Excluding foreign fishing access leads to similar average scores between high- and low-income EEZs (Lower plot **S**). Similar average scores are, however, explained by different mechanisms, namely excessive fishing capacity and subsidies in high-income EEZs and deficient scientific, political and enforcement practices in low-income EEZs.

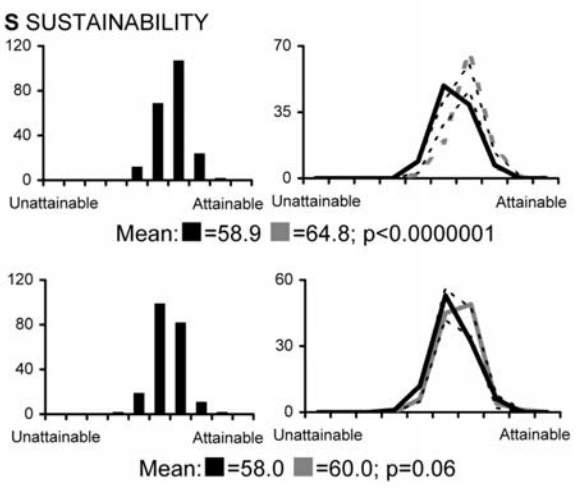

Supplement: Figure S1 — Variations in the number of countries with different qualities in their fishery management attributes. (0.48 MB PDF) [file pbio.1000131.s001.pdf]
