# Supplementary figures and images for: Management Effectiveness of the World's Marine Fisheries
Source: PLoS Biol. 2009 Jun 23;7(6):e1000131. doi: 10.1371/journal.pbio.1000131 (PMC2690453; doi:10.1371/journal.pbio.1000131)

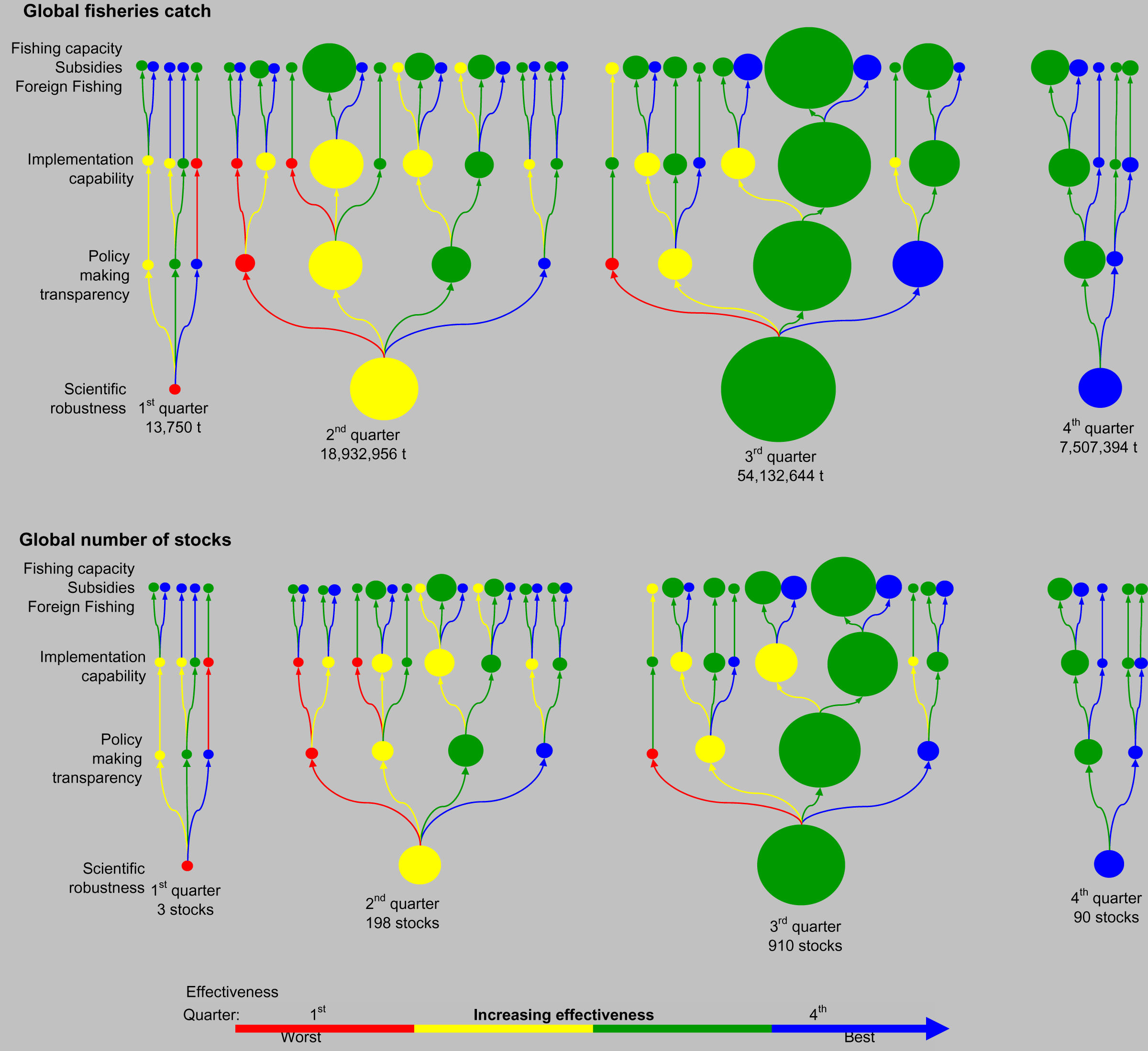

Supplement: Figure S2 — Discrimination of the world's fisheries catch and fished stocks according to different fishery management attributes. (0.84 MB TIF) [file pbio.1000131.s002.tif]

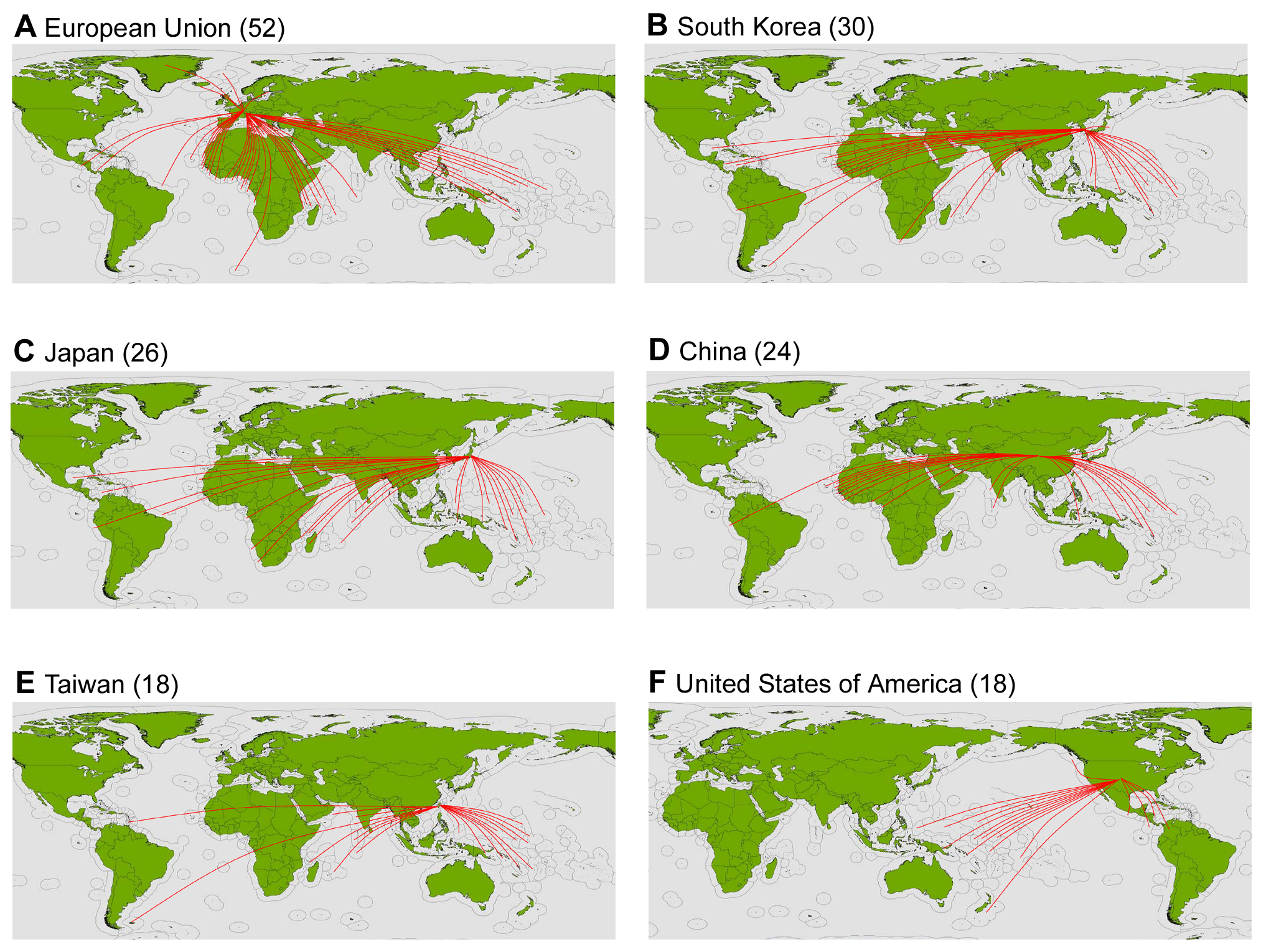

Supplement: Figure S3 — Countries with the largest use of foreign fishing access agreements. (9.31 MB TIF) [file pbio.1000131.s003.tif]

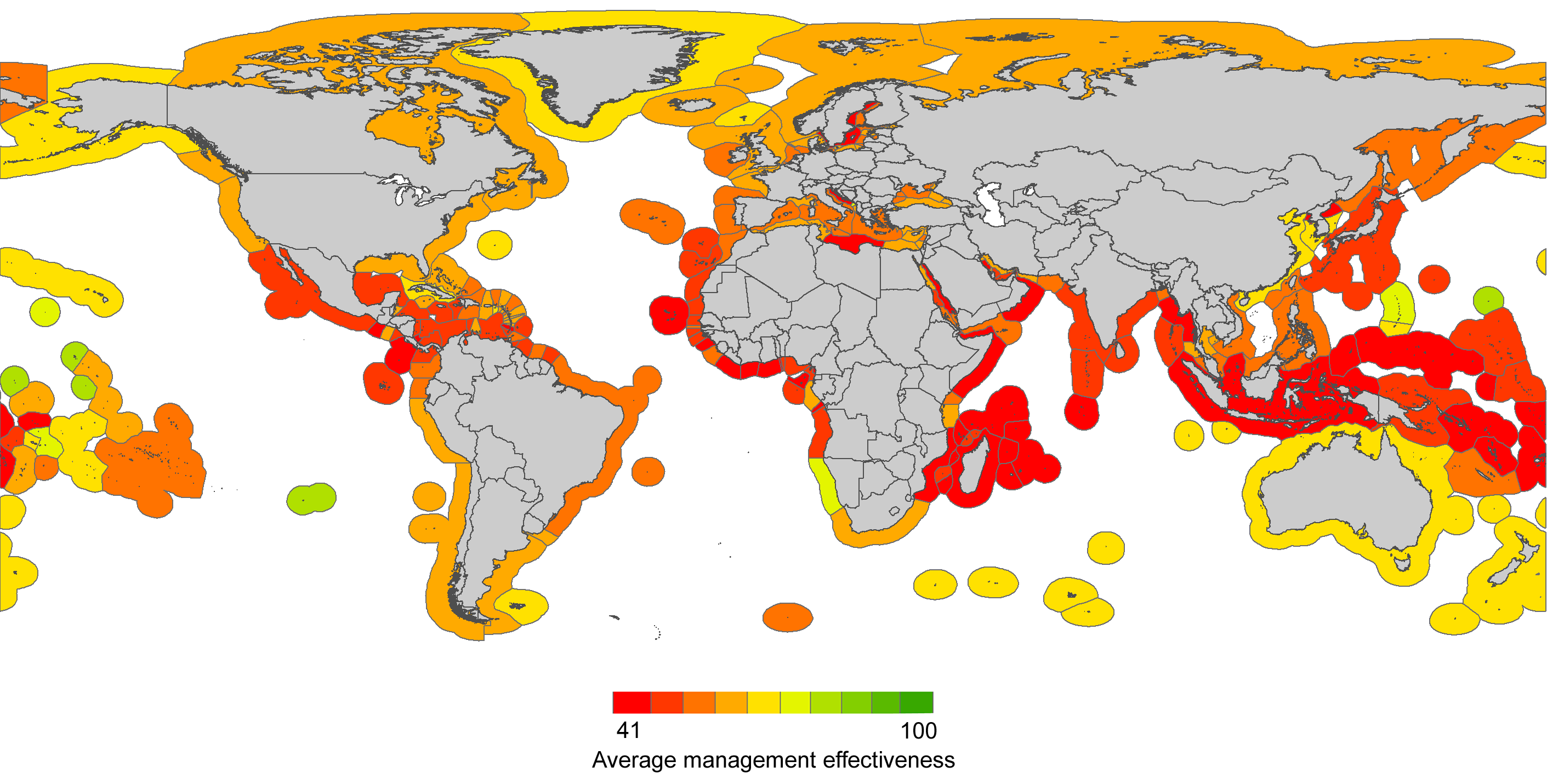

Supplement: Figure S4 — Global extent of recreational and small-scale fisheries and the frequency of countries imposing different types of regulations. (0.50 MB TIF) [file pbio.1000131.s004.tif]

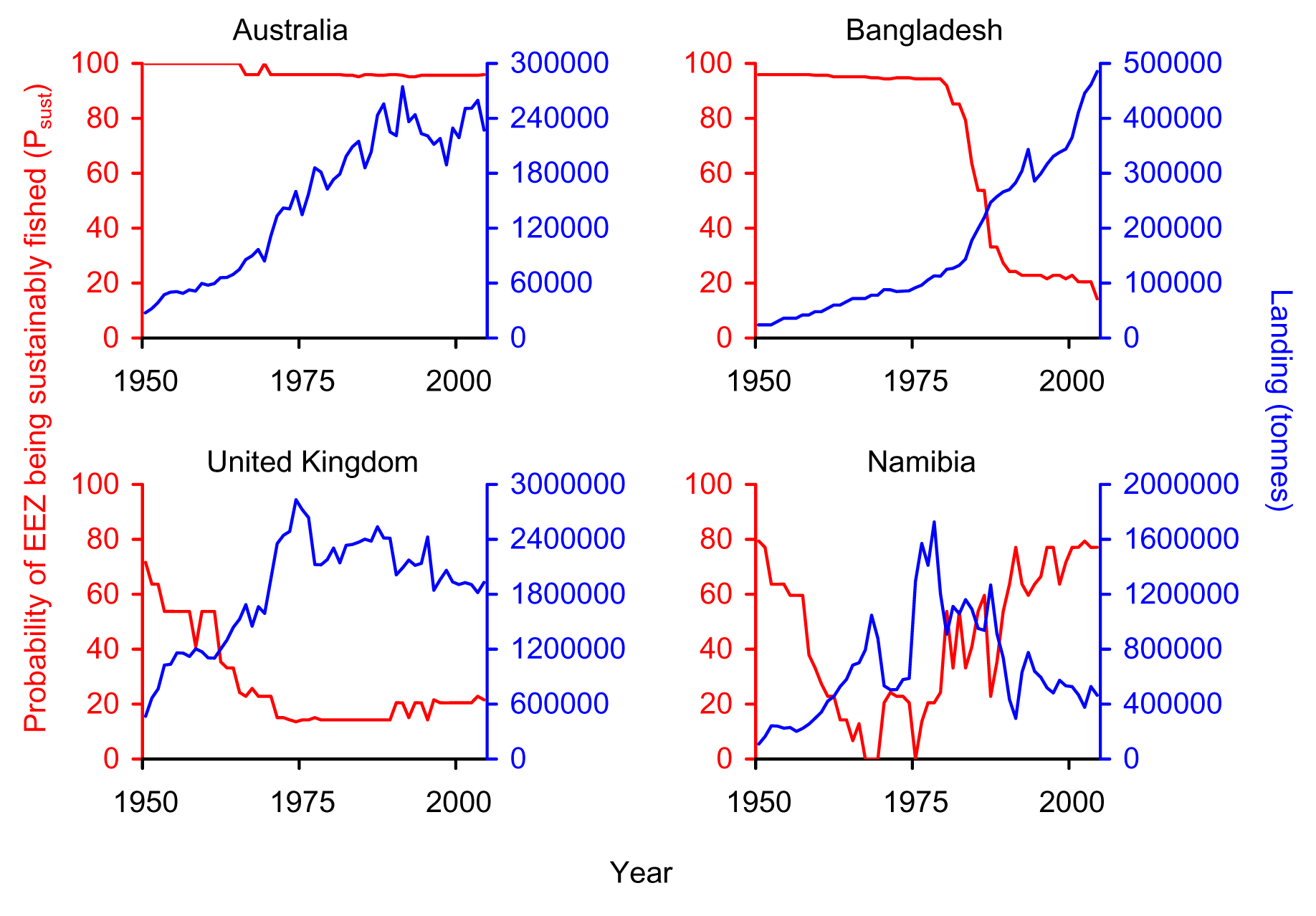

Supplement: Figure S5 — Robustness of the metric used to assess fisheries sustainability. (7.05 MB TIF) [file pbio.1000131.s005.tif]
