# Supplementary material for: Management Effectiveness of the World's Marine Fisheries
Source: PLoS Biol. 2009 Jun 23;7(6):e1000131. doi: 10.1371/journal.pbio.1000131 (PMC2690453; doi:10.1371/journal.pbio.1000131)
Supplement: Text S1 — Extended acknowledgements of the participants. (0.04 MB DOC) [file pbio.1000131.s006.doc]

**Text S1. Extended acknowledgements of the participants.** We are deeply grateful to all of those who participated in our survey and made this global audit possible. Three hundred and twenty-seven responders chose not to disclose their names. Those who did are listed below but cannot be linked to the results of any one country because in many instances we obtained duplicate responses for each country some of which may have been provided by national or international experts and because not all of those who participated chose to disclose their names; we make this statement to protect the integrity of participants in case of any discord. The names of those who participated in the survey and chose to disclose their names are: A W May; Arthur Jones; Abdillahi Omar Farah; Abdulla Naseer; Abdulmaula Hamza; Abigail Moore; Adrian Oviedo; Ahmedou Ould Mohamed El Moustapha; Aida D´Almeida; Aisake Batibasaga; Alaa Eldin Elhaweet; Alan White; Alassane Dieng; Alberto Fuentes Larenas; Alberto Halare; Alejandro Covarrubias; Alejandro Medina; Alejandro Pérez Velázquez; Aleksandar Joksimoviæ; Alex Brown; Alfonsina Romo; Ali Ismen; Alina González Flores; Alivereti Yaya; Alkaly Doumbouya; Ana L. Ibañez; Andres Hermida Trastoy; Andrew A. Rosenberg; Andrew Cohen; Andrew Field; Andrew Magloire; Andrew Syvret; Anesh Govender; Angel Ancona Ordaz; Angel Herrera; Ari Purbayanto; Arie Muhardy; Armagan Sabetian; Armando López Ramos; Asberr N.Mendy; Atanasio Brito; Audra Barrett; Augustine Mobiha; Babu Diwakar; Baldvin Baldvinsson; Bamy Idrissa Lamine; Barend Johannes Van Zyl; Being Yeeting; Ben Neal; Benjamin Toirambe; Bernardo Aliaga; Bill Holden; Bitoumba Andre; Bo Poulsen; Boedi Sardjana Julianto; Bolotova Natalia; Konovalov Alexander; Boodhun Ramcharrun; Borsa; Bourjea; Brian Johnson; Bruce M. Leaman; Budi Iskandar Prisantoso; Camilo B. García; Camilo Cuco; Carlos Augusto Borda Rodriguez; Carlos Garcia-Quijano; Carlos Godínez Reyes; Carlos Sanchez Plaza; Carlos Veloso; Carmen Kigimnang; Carole Ogandadas; Chabanet Pascale; Chand G.S.Devadawson; Changeux Thomas; Charles Perez; Chavance Pablo; Chien-Chung Hsu; Chita Guisado; Chris Heinecken; Christain Ramofafia; Christophe Brié; Chu Tien Vinh; Cirilo Vieira; Claudia Stella Beltrán; Cláudio Luis Santos Sampaio; Clive Fox; Clyde S. Tamaru; Cosme Caracciolo; Costas Papaconstantinou; Alexis Conides; Craig Bohm; Cristian Canales; D'Almeida Arsène F. M.; Daniel Caetano R. Inoque; Daniel Kosonoy Aceves; Daniele Bevacqua; David B. Mcclellan; David Kulka; David L. Allison; David Moe Nelson; David Williamson; Delfin Quezada Domínguez; Dirk C. Tijssen; Djibril Balde; Domba Félix; Domingo C. Flores Hernández; Domingo Ochavillo; Donald David; Donatella Del Piero; Alkaly Doumbouya; Ducrocq Manuel; Duncan Leadbitter; Ebrahim Abdulqader; Eduardo A. Mendoza Quintero Mármol; Eduardo Guillermo Pastor Rodríguez; Edward B. Bigler; Edwin Causado Rodriguez; Eirik Mikkelsen; Elizabeth Ann Mitchell; Eloy De Sousa Arújo; Eluri Apuchand; Elvira Ynion Adan; Emil Kuzebski; Emilio Michel Morfín; Delfin Quezada Domínguez; Enir Girondi Reis; Enrique Nuñez Lara; Eric Shaw; Erick Baqueiro Cardenas; Erling Bakken; Ernesto A. Chávez; Ernesto Enkerlin-Hoeflich; Ester C. Zaragoza; Eulogio Abeso Oyana; Eunice Perez-Sanchez; Evalds Urtans; F. Dilrosun; F. K. E. Nunoo; Fabian Blanchard; Fao Bernard; Fatma Sobo; Favreliere Philippe; Fedra Solano Chiriboga; Fernando Amestoy; Fernando Tuya; Fileonor O. Eleserio; Fini Aitaoto; Flomo P. Molubah; Andre Forest; Francesca Ottolenghi; Francisco Aguilar; Francisco Geraldes; Francisco José Gavidia Medina; Francisco Sardà; Franck Magron; Franz Mueter; Adel Gaamour; Gabriel Gómez Gómez; Garry Preston; Gbaguidi Amelie; Gellwynn Jusuf; Genoveva Cerdenares Ladrón De Guevara; Geoff Meaden; Geoffrey A. Matthews; Georgios Ioannou; Gerard Domingue; Giasuddin Khan; Gomal H. Tampubolon; Goryainov Alexander; Goujon Michel; Grant Leeworthy; Granvil D. Treece; Greg Ferguson; Greg Workman; Guillermo C Armas Bouza; Hans Jusseit; Hasan Yazlak; Hassan Kalombo; Henrik Sparholt; Henry De Cuba; Hjörtur Gíslason; Homayoun Hosseinzadeh Sahafi; Howel Williams; Hussein Abegaz Issa; Ian Knuckey; Ian Perry; Ibrahima Niamadio; Ignacio Sobrino Yraola; Imam Musthofa Zainudin; Indar Ramnarine; Ingo Wehrtmann; J. A. Clark; J. Adán Caballero Vázquez; Jagath Wickrama Rupasinghe; James C Gumbs; James M. Bishop; Mohsen Al-Husaini; Jane Mbendo; Javier Jacinto Archbold Hawkins; Javier Villegas Sierra; Jawahar Paulraj; Jean Robert Badio; Jean Wiener; Jesus Marcano; Jim (James) Portus; Jimena Bohórquez Herrera; João Correia; João Pereira; Joaquin Buitrago; Jocemar Tomasino Mendonça; Joel Nageon De Lestang; Johann Augustyn; John Bothwell; John Isaksen; John L. Munro; John Rice; Johnson U. Kitheka; Jon Mar Halldorsson; Jonathan O. Dickson; Jorge Enrique Paramo; Jorge Viaña Tous; José Alejandro Rodríguez Valencia; Jose Alio; José Augusto Negreiros Aragão; Jose Eduardo Perez Catzin; José Francisco Lazo Marín; Jose Francisco Torres; Jose Ignacio Fernandez Mendez; Jose R. Cañón; Joseph Paul Kavalam; Jouker; Joyce Samuelu Ah-Leong; Juan Carlos Chavez Comparan; Juan Carlos Gonzalez; Juan Freire; Juan L. Maté; Julia Jimenez Rodriguez; Julian Bauer; Julio A. Sanchez Chavez; Julio F. Chocca; Julio Harvey; Jung-Hee Cho; K. Ponnusamy; K. Sakkaravarthi; Kadir A Mohamud; Kali-Tchikati Edouard Et Atsango Benoît Claude; Karl Kr. Angelsen; Keith Granger Davis; Ken Leber; Kinibo Ura; Kirstie Knowles; Kjell Nedreaas; Kumbi Kilongo Nsingui; Kungwan Juntarashote; Kuo-Nan Chung; Philippe Laleye; Lance Morgan; Le Fur; Le Ry Jean Michel; Leban Gisawa; Lennin Florez- Leiva; Leslie Joseph; Linda Lombardi-Carlson; Llena Sang García (Varón); Lorcan O Cinneide; Lourdes Jiménez Badillo; Lubna Hamoud Al-Kharusi; Lucien Dehy; Luis Castro U; Luis Eduardo Briceño; Luis O. Duarte; Luis Villegas; Luisa Muñoz; M J Ball; M. Ben-Yami; Carlos Solis Gil; M. S Adam; M. Wasim Khan; M.R. Boopedranath; Ma. Eugenia Vega Cendejas; Mads Trolle Nedergaard; Magese Emmanuel Bulayi; Maia Metreveli; Maja Fredotovic; Malouli Idrissi Mohammed; Manal R. Nader; Manoj Nair; Manuel Haimovici; Manuel Mendoza Carranza; Manuel Oliva; Manuela Bandeira; Marc Gagnon; Marcela Zamorano; Marceliano; Marco L. Bianchini; Marcus Henrique Carneiro; Margarita Cervantes Trujano; Maria A. Gasalla; María C. Valdez; Marìa Del Carmen García Rivas; Marilu Bouchon Corrales; Mario Israel Rojo Amaya; Marius Diemont; Mark Dimech; Mark Showell; Martha Beatriz Rendón López; Martha Rivera García; Maruia Kamatie; Marwan; Mary Ann Palma; Mary Labropoulou; Charles Massa; Mathew Chigiyal; Maurice Brownjohn; Mauro Gongora; Maximiliano Bello; Menakhem Ben-Yami; Michael Lowry; Michael S. Trianni; Michael Tosatto; Michaela Aschan; Michelle Scharer; Miguel Chancerelle De Machete; Miguel Ortega; Mike King; Mike Pawson; Mimoza Cobani; Mitsutaku Makino; Mohamed Abdullah Saasd Hasan; Mohamed M. Abou Zaid; Mohamed Shainee; Mohammad A. Saif Abdullah; Mohammad Pourkazemi; Mokhtar Akhondi; Mose Pelasio; Muhamad Saini B. Syluansa; Muhammad Ali Shah; Mwakio Tole; N. Dilyaur Malsol; N.A.Basheer Ahamed; Nabil A. A. Al-Shwafi; Narriman Jiddawi; Nasim Akhtar; Naviti William; Nfamara J. Dampha; Ngoande Salvador; Ngwe Assoumou Christian; Nicolàs Castañeda Lomas; Nicos Hadjistephanou; Nikita Gabor; Njifonjou Oumarou; Nohora Galvis; Carole Ogandagas; Olavi Kaljuste; Oliver R.T. Paderanga; Marcel Ordan; Oscar Daniel Pin; Oscar Sosa-Nishizaki; Osman Mohamed Saeed; Ousman Mass Jobe; Oystein Hermansen; P.E.Cheran; P.O.J. Bwathondi; Pablo Granados-Dieseldorff; Palladin; Pasience Magoha; Patricia Arceo; Patricia Madrigal Cordero; Patricia Toledo; Patrick S. Fong; Paul E. Phillip; Pedro Saenz Martinez; Peter A. Murray; Peter H. Flournoy; Peter Mous; Petros Chigwechokha; Philip Leonard Cadwallader; Philip Miller; Philippe La Hausse De Lalouviere; Philippe Lallemand; Pio Manoa; Praulai Nootmorn; J.B. Okeyo-Owuor; Radu Suciu; Rafael Chavez Lopez; Ralf Döring; Ramon De Leon; Randriamahazo Herilala; Rasolonjatovo Harimandimby; Ravi Fotedar; Raymond Blake; Renaldi Barnutty Navarro; Renato Azevedo; Matias Silvano; René Schärer; Ricardo J. Haroun; Ricardo Torres Lara; Richard Donald Braley; Richard Dudley; Richard Inurritegui; Rocío Tíjaro Rojas; Roland Azemia; Rory Campbell; Rosalie Masu; Roy Melville-Smith; Rui De Paula E Silva; S. M. Nurul Amin; S.Balu; S.K. Chakraborty; Sahar Fahmy Youssif Mehanna; Samliok Ndobe; Samuel Juarez; Samuel Ramos Carrillo; Sanja Matic Skoko; Sanja Matic-Skoko; Scott Monks; Sedzro Kossi Maxoe; Senthil Kumar; Seraphin Dedi Nadje; Sergio Garcia; Sergio Hoare; Sergio Joel Niebla Rodriguez; Sergio Martínez C.; Shep Helguile; Shyama Rathacharen; Sidibe Aboubacar; Simion Nicolaev; Simon Bossy; Sione Vailala Matoto; Slam Kelen; Sonja Teelucksingh; Soule Hamidou; Soumah Mohamed; Stella Williams; Stephan Gollasch; Steve Hall; Stuart Beaton; Suriyan Vichitlekarn; Susan Singh-Renton; Susan Waugh; Svein A. Iversen; Sylla Ibrahima Sory; T Mahidhar Chowdary; Tahilandé Barreto Zavala; Tejnarine Shawn Geer; Telmo Morato; Terry Keju; Terry Smith; Thamasak Yeemin; Thomas Kocherry; Tom Kompas; Tony W Taleo; Tran Xuan Loi; Tuilava S Uota; Tunesi Leonardo; Ulf Bergström; Vahdet Unal; Vassen Kauppaymuthoo; Vassily Spiridonov; Vaughn Andrew Charles; Venancio Guedes De Azevedo; Verhegghen J-Fr; Verónica Castañeda Fernández De Lara; Vicente Anislado-Tolentino; Victorino Nahada; Vijoy Joseph; Vilma Correa; Virginia Garrison; Vivienne Solís Rivera; Vladimir Radchenko; Volker Siegel; Walain Ulaiwi; Warren Papworth; Wayne Chen (Wen Chen); Will Zacharin; Wim Demaré; Wojciech Pelczarski; Xiaorong Zou; Yevewuo Z. Subah; Yolanda Babb; Yongwen Gao; Yugraj Singh Yadava; Zoran Spirkovski.
